# Intraoperative elastography and spinal surgery: a systematic review of current and future applications in clinical and preclinical models

**DOI:** 10.1186/s13089-025-00462-0

**Published:** 2025-11-19

**Authors:** Parker Dhillon, Brian Fabian Saway, Audrey Galimba, Rishishankar Suresh, Thomas Eckert, Max J. Kerensky, Vikas N. Vattipally, Patrick Kramer, Nicholas Theodore, Sunil Patel, Stephen Kalhorn

**Affiliations:** 1 Department of Neurosurgery, Medical University of South Carolina, 96 Jonathan Lucas St #307, Charleston, SC 29425 USA; 2 Department of Neurosurgery, Johns Hopkins University School of Medicine, Baltimore, MD USA; 3 Department of Biomedical Engineering, Johns Hopkins University School of Medicine, Baltimore, MD USA; 4 Department of Orthopaedic Surgery, Johns Hopkins University School of Medicine, Baltimore, MD USA

**Keywords:** Ultrasound elastography, Spinal pathology, Intraoperative ultrasound, Surgical imaging

## Abstract

**Background:**

While conventional imaging provides excellent structural detail of the spine, it cannot assess the mechanical properties of spinal tissue in real time. Ultrasound elastography (USE) is an emerging modality that quantifies tissue stiffness, offering a potential solution to this diagnostic gap. This review synthesizes the current evidence for the use of USE in spinal pathology.

**Main body:**

A systematic review of the PubMed, Cochrane, and Web of Science databases was conducted in accordance with PRISMA guidelines, yielding seven primary studies, three clinical and four preclinical, published between 2015 and 2024. These studies, comprising preclinical and clinical data, demonstrate USE's ability to provide real-time, quantitative feedback. Key applications identified include quantifying tension relief in tethered cord syndrome, differentiating spinal tumors from healthy tissue based on stiffness values, and assessing the biomechanical severity of acute and chronic spinal cord injury. Shear wave elastography (SWE) was the predominant modality, proving superior to strain elastography (SE) for spinal applications.

**Conclusion:**

USE is a powerful adjunct to traditional spinal imaging, providing unique functional data that can enhance intraoperative surgical precision and decision-making. While challenges such as depth penetration and operator standardization remain, continued research and technological innovation position USE to significantly improve diagnostic accuracy and surgical outcomes in spinal disease management.

**Supplementary Information:**

The online version contains supplementary material available at 10.1186/s13089-025-00462-0.

## Introduction

Pathologies affecting the spine and spinal cord are highly prevalent and represent a significant source of morbidity and disability worldwide. Conditions such as traumatic spinal cord injury, degenerative cervicothoracic myelopathy, spinal cord tumors, and tethered cord syndrome can lead to profound neurological deficits, impairing motor, sensory, and autonomic functions [1]. Given their potential to cause irreversible damage without appropriate surgical intervention, timely and accurate diagnosis is critical to optimizing patient outcomes in these scenarios.

Current imaging techniques such as magnetic resonance imaging (MRI) and computed tomography (CT) provide excellent structural detail, but are not currently configured to assess the mechanical properties of spinal tissue in real time [2]. Meanwhile, vessel and perfusion-based modalities like diagnostic spinal angiograms and contrast-enhanced ultrasound (CEUS) offer vascular insights but do not provide information on tissue stiffness in evaluating spinal conditions such as tethered cord syndrome, cervicothoracic myelopathy, tumors of the spinal cord, and inflammatory process of the spinal cord [3].

Ultrasound elastography (USE) is an emerging imaging modality that provides real-time, quantitative assessment of tissue stiffness, initially developed for hepatology and now expanding into neurosurgery [4, 5]. It is particularly valuable in spinal pathology, where it aids in assessing compression, tracking disease progression and evaluating surgical outcomes [4]. The two primary types of elastography are strain elastography (SE), which relies on tissue deformation for qualitative stiffness maps, and shear wave elastography (SWE), which measures stiffness in kilopascals (kPa) for more reproducible, objective measurements [6, 7]. SE, which relies on tissue deformation from manual compression, is heavily operator-dependent and has a limited role in spinal applications where the cord is not amenable to compression. In contrast, SWE utilizes an acoustic radiation force impulse to generate shear waves, quantifying stiffness based on their propagation speed. Given its ability to provide reproducible, operator-independent measurements in kPa without direct compression, SWE has emerged as the preferred and more technically feasible modality for neurosurgical applications.

Previous systematic reviews have explored the broad applications of elastography in neurosurgery. For instance, Hersh et al. 2022 conducted a wide-ranging review of operative neurosurgery, identifying only two studies related to the spine, while Albakr et al. 23 also provided a broad overview with only a single spinal study included. Other reviews on the general use of intraoperative ultrasound in spine surgery, such as Ganau et al., have highlighted its utility for confirming decompression and guiding instrumentation, noting elastography as a promising, future-facing development [8]. More recently, Ali et al. reviewed the technical aspects of intraoperative ultrasound for spinal cord injury and a 2025 AO Spine consensus paper recommended standard IOUS to confirm decompression, but neither focused specifically on the modality of elastography [9, 10]. While these reviews provide valuable context, a dedicated synthesis of evidence for the specific use of ultrasound elastography in spinal pathology is currently lacking. This review aims to fill that gap.

Challenges like limited depth penetration and operator familiarity remain barriers to widespread clinical adoption [11, 12]. While USE is not yet a standalone diagnostic tool, its integration with other imaging methods offers valuable real-time insights into spinal pathology. More large-scale trials and standardization are needed to fully realize its potential in neurosurgical practice [7, 13]. Therefore, this systematic review aims to evaluate the diagnostic and prognostic utility of USE for a range of spinal pathologies—including tethered cord syndrome, spinal tumors, and spinal cord injury—and to assess the evidence for intraoperative USE as a tool for enhancing surgical guidance, quantifying interventional success, and improving real-time surgical decision-making. Ultimately, this review synthesizes the current evidence to inform clinical practice and define key directions for future research in the application of elastography in spinal neurosurgery.

## Methods

### Search criteria

A systematic review was conducted to evaluate the application of USE in spine and spinal cord pathology. This review was written according to the PRISMA 2020 guidelines [14]. The primary searches were performed in PubMed, Cochrane Library, and Web of Science for studies published between January 2015 and January 2025. A comprehensive search strategy was developed in consultation with the author team to identify all relevant studies, and the full electronic search strings for each database are detailed in Supplementary Table 1. The search was conducted in January 2025. Only peer-reviewed studies, clinical trials, and systematic reviews published in English were included. To ensure a comprehensive review, a supplementary search was also conducted, which included screening the Scopus database for grey literature (conference abstracts, book chapters) and hand-searching the reference lists of included articles.

Two independent reviewers (PD and AG) screened titles and abstracts for relevance, followed by full-text reviews of eligible articles. Disagreements were resolved by discussion. Data extracted from each study included sample size, elastography modality, clinical outcomes, key findings, and potential future applications.

### Study risk of bias assessment

To assess the methodological quality of the included non-randomized studies, three reviewers (PD, AG, and BS) independently evaluated the risk of bias using the Risk Of Bias In Non-randomized Studies—of Interventions (ROBINS-I) tool [15]. The tool assesses bias across seven domains: confounding, selection of participants, classification of interventions, deviations from intended interventions, missing data, measurement of outcomes, and selection of the reported result. Disagreements between reviewers were resolved through discussion to reach a consensus.

While the ROBINS-I tool was designed primarily for human studies, it was applied to all primary research, including animal models, to ensure a standardized assessment framework across studies. It is acknowledged that specialized tools for animal studies exist, and potential differences in the nature of bias between clinical and preclinical studies were considered during interpretation.

### Data synthesis

Given the significant heterogeneity in the included studies with respect to patient populations, study designs, and specific elastography techniques used, a statistical meta-analysis was deemed inappropriate. Therefore, a structured narrative synthesis of the findings was performed, with results grouped thematically by study subjects, interventions applied, clinical application, and potential future directions. A summary of findings from the included literature is presented in Table 1. The key advantages identified for each major pathology are also synthesized in Table 2**.**

**Table 1:** Summary of literature on Ultrasound Elastography (USE) and Shear wave elastography (SWE) on spinal applications and potential uses

Authors	Participant Type	Center(s)	Participants	Interventions	Primary Findings
Al-Habib et al. (2021) [15]	Human	King Saud University	N = 25	Used SWE to compare spinal cord elasticity in patients with compressive lesions versus those with adequate decompression after surgical intervention	Spinal cord elasticity is significantly increased in patients with compressive lesions compared to lesions with adequate decompression after laminectomy or corpectomy
Al-Habib et al. (2018) [23]	Canine	King Saud University	N = 10	Measured spinal cord elasticity using SWE in dogs subjected to mechanical loads via balloon compression	USE feasibly measures spinal cord stiffness intraoperatively, characterizing a linear relationship between compression load and elasticity. Demonstrates applicability for monitoring spinal cord health
Almotairi et al. (2023) [18]	Human	King Saud University	N = 1	Evaluation of spinal cord stiffness pre- and post-untethering surgery using ultrasound elastography	Post-untethering, spinal cord stiffness decreased significantly, demonstrating SWE’s ability to quantify surgical outcomes
Kerensky et al. (2024) [16]	Human + Cadaver	Johns Hopkins	N = 6 human surgeries, 1 Cadaver	Computational and intraoperative studies using elastography assessed tethered spinal cord tension pre-treatment	Established a linear relationship between tethered spinal cord tension assessed using computational and squared SWE measurements. Posited USE as a potential physical, clinical measure of spinal cord tension
Prager et al. (2020) [22]	Canine	University of Bristol	N = 3	Analysis of spinal cord stiffness utilizing USE for developing stiffness-matched biomaterials for SCI	USE provides a noninvasive measure for stiffness-matched biomaterials for SCI treatment
Shajudeen et al. (2019) [24]	Rabbit (ex vivo)	Texas A&M University	N/A	Employed elastographic axial strain modeling for identifying spine fractures in ex vivo rabbit spines	Elastography axial modeling aided in detecting a wide variety of spinal fractures, offering a non-invasive diagnostic tool for pre- and postoperative analysis
Tang et al. (2023) [25]	Rabbit (in vivo)	Houston Methodist	N = 11	Evaluation of spinal cord injury severity using SWE in a rabbit model, with implications for human translation	SWE accurately assessed spinal cord injury severity among subjects (p < 0.05), suggesting potential for clinical applications in human SCI diagnosis

**Table 2:** Potential advantages of ultrasound elastography in spinal pathologies

Pathological condition	Potential advantage(s)	Modality used	Key supporting citations
Tethered Cord Syndrome (Clinical)	• Real-time, quantitative feedback on cord tension • Immediate confirmation of surgical success • May reduce revision surgeries	SWE	Kerensky et al. (2024), Judy et al. (2023), Almotairi et al. (2023)
Spinal Oncology (Clinical)	• Differentiates pathologic from healthy tissue • Potential to improve gross-total resection • Quantitative basis for tumor margins	SWE	Al-Habib et al. (2021)
Acute Spinal Cord Injury (Preclinical)	• Immediate, real-time assessment of injury severity • Correlates stiffness/strain with functional outcomes • Potential early biomarker	SWE & SE	Prager et al. (2020), Al-Habib et al. (2018), Shajudeen et al. (2019), Tang et al. (2023)
Chronic Spinal Cord Compression (Clinical)	• Quantifies elevated stiffness in compressed cords • Stiffness reduction correlates with neurological improvement • Potential prognostic tool for recoverability	SWE	Al-Habib et al. (2021)

This table summarizes the key advantages of USE for each pathological condition identified in this systematic review. The findings are categorized by pathology and list the primary elastography modality used, along with the key supporting citations from the included studies

*SWE* shear wave elastography, *SE* strain elastography

### Inclusion and exclusion criteria

Studies were eligible if they examined USE in assessing spinal cord pathology, focused on clinical applications or in vivo models, and reported quantitative elastography findings such as stiffness measurements, diagnostic performance, or surgical outcomes. Exclusion criteria included studies focused on magnetic resonance elastography, muscle or fascia stiffness investigations without direct spinal cord relevance, feasibility studies without clinical correlations, and articles lacking methodological rigor or peer-review.

## Results

**Fig. 1:**
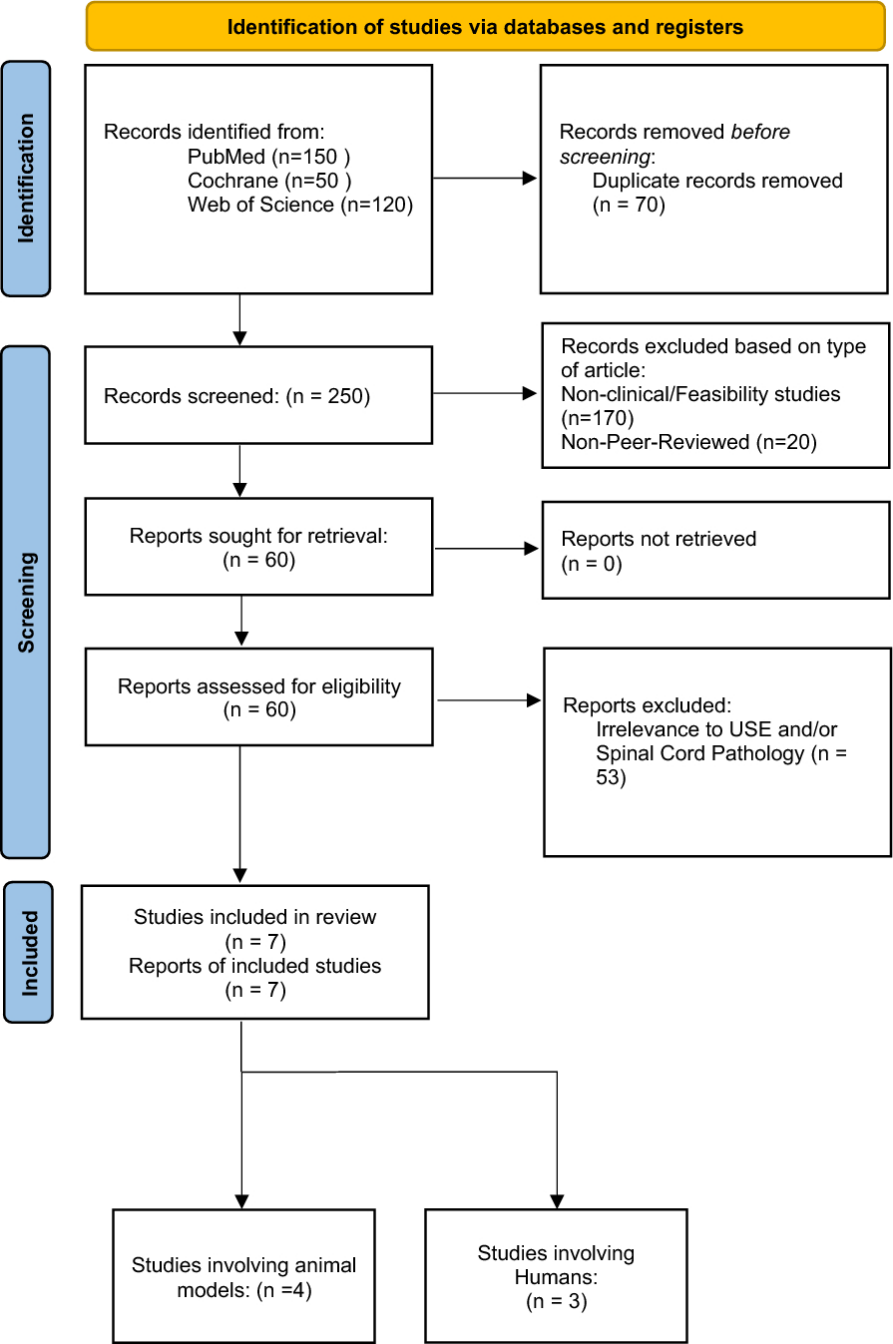
PRISMA flow diagram for review, detailing database searches, records screened, and studies spanning modalities included

The initial search yielded 320 articles, with 25 records identified from secondary sources. After removing 70 duplicates, 250 articles underwent title and abstract screening. 170 studies were then screened out for article type and 20 studies were further excluded as not being peer reviewed, either being redundant abstracts or available as pre-print. Of the 60 full-text articles reviewed, 53 were excluded for reasons including focus on magnetic resonance elastography (n = 5), emphasis on muscle or fascia stiffness without direct spinal cord relevance (n = 9), and further lack of clinical data and patient sample size (n = 39). Ultimately, 7 studies met the inclusion criteria and were included in the final analysis, as outlined in the PRISMA flow diagram **(**Fig. 1**)**. Our supplementary search of the Scopus database and other secondary sources did not identify any additional unique, peer-reviewed studies that met the inclusion criteria.

The included studies, published between 2015 and 2024, involved a cumulative sample size of 57 subjects, with individual study sizes ranging from case studies of 1 patient to case series of 25 participants. The studies explored USE in humans (n = 3) and in animal models (n = 4. Notably, with some overlap, applications of USE identified in these studies included the assessment of tethered spinal cord syndrome (n = 4), postoperative monitoring of spinal scar tissue (n = 3), intraoperative applications for spinal tumors (n = 2), and quantitative stiffness evaluation in chronic spinal conditions (n = 2).

**Fig. 2:**
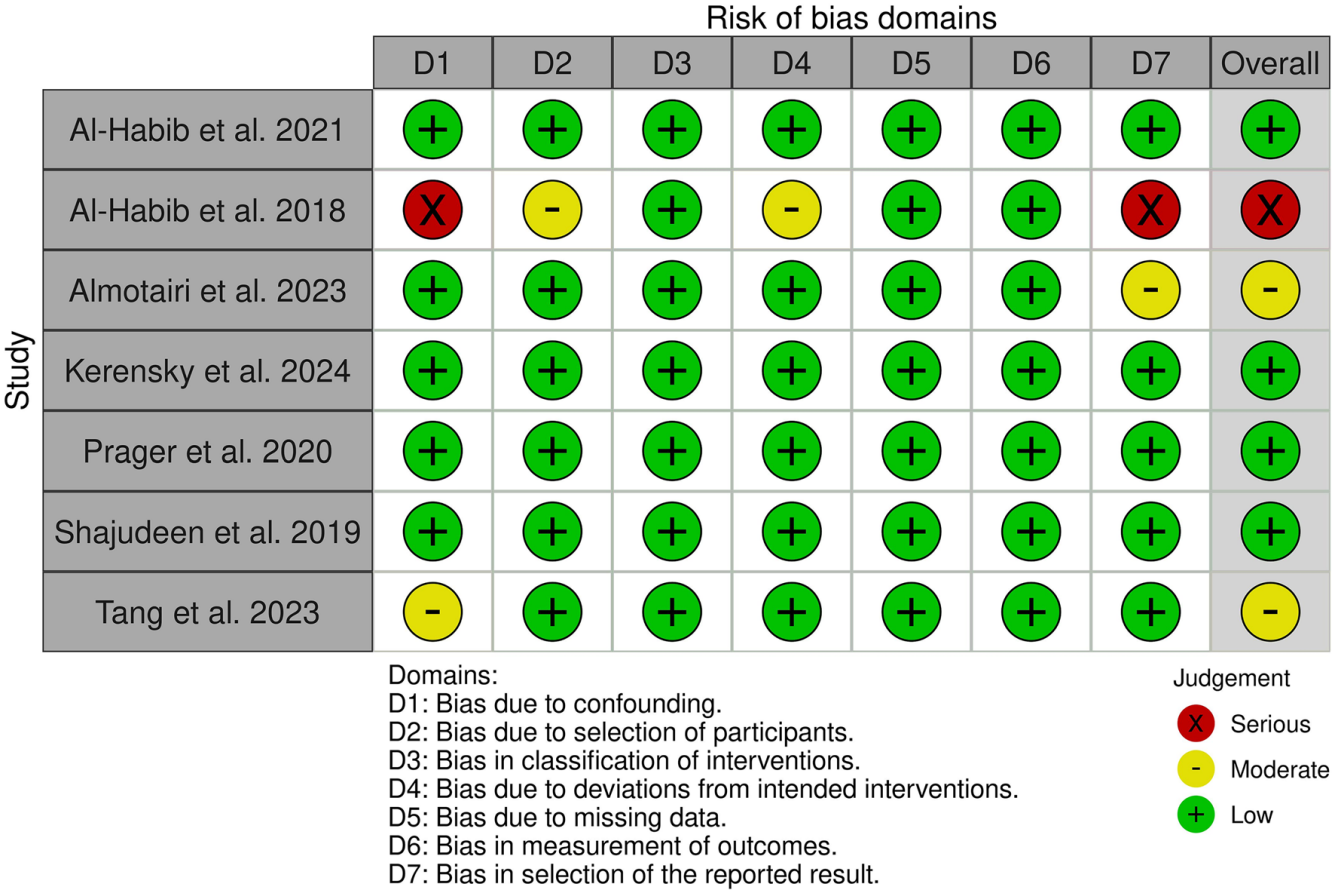
Summary of Risk of Bias Assessment. The traffic light plot summarizes the risk of bias judgments for each included study using the ROBINS-I tool. Each column represents a specific bias domain, and the final column provides the overall judgment. Green indicates a low risk of bias, yellow indicates a moderate risk, and red indicates a serious risk

The methodological quality of the included primary studies was assessed using the ROBINS-I tool, with findings summarized in Fig. 2. Of the seven primary studies evaluated, the overall risk of bias was found to be low to moderate. Four studies were judged to be at a low risk of bias, two were at moderate risk, and one was found to have a serious risk of bias. The moderate risk ratings were primarily due to potential bias in confounding and in the selection of reported results. The single study with a serious risk of bias showed significant concerns in the same domains, in addition to bias in participant selection and deviations from the intended intervention. No studies were found to be at a critical risk of bias. Detailed assessments for each included study are provided in Supplementary Table 2.

## Discussion

### Clinical applications of elastography in spinal surgery

USE measures tissue stiffness in real time, providing valuable quantitative data for both preoperative evaluation and intraoperative decision-making. USE consists of two primary clinical modalities: SE and SWE. SE relies on tissue deformation under manual or physiological compression, generating qualitative stiffness maps. However, its accuracy is heavily operator-dependent and less reliable for deeper structures due to ultrasound signal attenuation [16]. Additionally, it has a limited role in spinal cord pathology as the spinal cord is not amenable to manual compression due to risk of injury. This specific limitation of applying external compression to the delicate spinal cord has been noted in prior reviews [8]. However, no study has investigated the risk of spinal cord injury from SE imaging.

In contrast, SWE generates mechanical shear waves via an acoustic radiation force impulse and quantifies tissue stiffness based on wave propagation speed. Stiffer tissues allow faster wave transmission, providing reproducible and near operator-independent measurements, though factors like transducer pressure and angle can introduce variability [6]. Given its ability to assess deeper structures accurately without requiring direct manual compression, SWE has become the preferred elastography method in neurosurgical applications. Compared to MRI elastography (MRE), USE also offers several advantages, including real-time imaging, portability, and intraoperative applicability, which MRE lacks due to longer acquisition times and the need for specialized equipment [17, 18]. Integrating USE into standard neurosurgical workflows provides surgeons with functional data that supplements traditional imaging modalities.

Initially developed for liver fibrosis assessment, elastography has been adopted across multiple medical fields, including musculoskeletal imaging, oncology, and neurosurgery [4, 5]. These intraoperative capabilities provide mechanical insights that enhance surgical precision with minimal disruption to workflow. In the field of hepatology, USE has demonstrated the ability to distinguish pathological from healthy tissues, improving the accuracy of diagnostic imaging and tumor resections [19]. However, the clinical application of USE is not without challenges. Despite advancements in technology, depth penetration remains a limitation, as ultrasound signal attenuation significantly affects imaging accuracy in deeper spinal structures [6]. Additionally, operator variability in SE introduces inconsistencies, underscoring the need for standardization in clinical use. [12]

While USE is a powerful adjunct to existing imaging modalities, its full diagnostic and prognostic potential for spinal pathologies remains under investigation. Large-scale trials and standardized protocols across pathology treatments are essential for enhancing reproducibility and expanding its role in neurosurgical practice [7]. This review of available literature culminates and synthesizes the work of the many investigators that have explored the applicability and efficacy of USE for pathologies of the spine and spinal cord. A summary of the available literature discussed can be found in Table 1.

### Tethered cord syndrome

#### Clinical studies

Tethered cord syndrome is a condition where excessive spinal cord tension leads to neurological deterioration. In this condition, studies have demonstrated that SWE effectively quantifies tension relief levels intraoperatively. Preliminary results of Kerensky et al., initially explored by Judy et al., explored a case series in which SWE was used to measure spinal cord tension pre- and post-surgical untethering via VCS [20, 21]. Their findings confirmed that SWE quantified surgical success which was further corroborated by Almotairi et al., who demonstrated a statistically significant reduction in spinal cord tension post-surgery. [22]

**Fig. 3:**
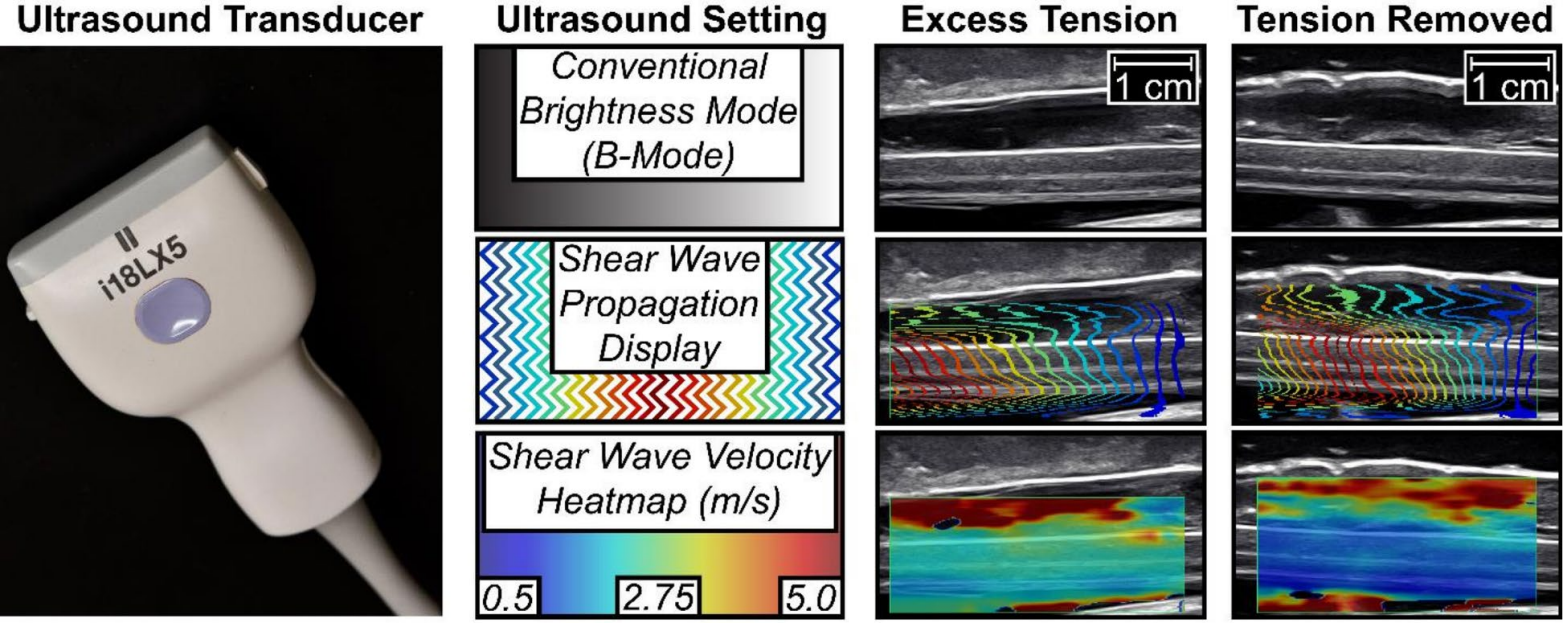
The promising utility of elastography to intraoperatively differentiate the physical state of a patient’s spinal cord is shown. Here, a posterior vertebral column subtraction osteotomy (PVCSO) was performed to treat tethered cord syndrome (TCS) by alleviating excess tension across the spinal cord. In this case, an i18LX5 ultrasound transducer attached to an Aplio i800 (Canon Medical Systems, Otawara, Japan) was used to capture the data. This figure is adapted from Kerensky et al. (2024), "Tethered spinal cord tension assessed via ultrasound elastography in computational and intraoperative human studies," Commun Med, CC BY 4.0 (https://doi.org/10.1038/s43856-023-00430-6). Components from Fig. 4 in Kerensky et al. (2024) have been re-labeled and repositioned with the addition of a supplemental image of the transducer for succinctness and clarity. (See: https://creativecommons.org/licenses/by/4.)

 Kerensky et al*.* then finalized this concept by utilizing shear-wave elastography (SWE) to assess changes in intraoperative stiffness during vertebral column shortening (VCS) [20]. Following surgical exposure of the spinal cord, which provides an ideal acoustic window, SWE was utilized to acquire stiffness data before and after the untethering maneuver. By exposing the entire spinal cord, this technique provided an optimal setting for SWE. Figure 3 illustrates this application during a posterior vertebral column subtraction osteotomy (PVCSO) for tethered cord syndrome, demonstrating the reduction in spinal cord stiffness following untethering, as adapted from Kerensky et al. [20]. Their findings demonstrated a significant reduction in spinal cord stiffness following VCS. Additionally, a linear relationship was established between cord tension and squared shear wave velocity measurements, supporting USE as a clinically relevant marker for spinal cord tension and successful operative intervention.

 These results highlight the potential for intraoperative SWE to augment surgical decision-making by offering immediate feedback on the state of the spinal cord ensuring adequate tensile reduction and potentially reducing the need for revision surgeries. SWE may help identify patients at higher risk for persistent deficits and guide early rehabilitation strategies.

### Spinal oncology

#### Clinical studies

USE has shown significant promise in differentiating malignant from benign tumors in other fields such as hepatology [5]. For example, SWE measurements above 5 kPa in hepatic elastography have been associated with a significantly higher likelihood of malignancy [23]. The adoption of USE into neurosurgery has been slow, but initial studies have shown promising results in assessing spinal tumors.

The work by Al-Habib et al. 2021 assessed spinal cord elasticity using intraoperative SWE in 25 patients with compressive lesions, six of which were spinal tumors (three meningiomas, one chondrosarcoma, one conus-area extramedullary tumor and one ependymoma). Their findings showed that the elasticity values for these tumors ranged from 30 to 128 kPa, which was substantially stiffer than the decompressed spinal cord (approximately 6.5 to 9.35 kPa). This stark contrast suggests that USE demonstrates the ability to differentiate pathologic from healthy spinal tissue based on significant differences in stiffness values, providing a quantitative basis for defining tumor margins. Chan et al. even reported that SWE outperformed surgeons in detecting residual tumor tissue, with a 94% sensitivity compared to 36% by the operating surgeon alone, underscoring its potential role in improving gross-total resections. [5]

Other systematic reviews by Hersh et al*.* and Albakr et al*.* have summarized the stiffness values for various brain tumors. Their findings indicate that primary tumors like intracranial meningiomas (approximately 33.1 kPa) are generally stiffer than metastatic tumors (16.7 kPa) and that low-grade gliomas are often stiffer than high-grade gliomas (11.4 ± 3.6 kPa vs 23.7 ± 4.9 kPa.) [4]. The stiffness values for spinal meningiomas (ranging up to 128 kPa) reported in the primary literature appear considerably higher than their intracranial counterparts, a finding that warrants further investigation. Furthermore, a review by Ali et al*.* highlighted the general potential of intraoperative ultrasound to help define tumor margins [10]. These established benchmarks for brain tumors provide a valuable reference point and confirm that similar principles of tissue characterization can be applied and refined for spinal oncology.

Future research should focus on establishing standardized stiffness thresholds across different spine and spinal cord tumor types, similar to existing benchmarks in hepatic and breast elastography, to further enhance the diagnostic and prognostic value of SWE in spinal oncology.

### Acute spinal cord compression and injury

#### Preclinical models

Acute spinal cord injury (SCI) typically results from sudden trauma, causing rapid biomechanical and vascular disruption that can lead to irreversible neurological damage. In this setting, elastography offers the potential for immediate injury severity assessment. Elastography has emerged as a valuable tool in animal and clinical models to provide real‐time assessments of these abrupt mechanical changes. In this context, Prager et al. offer critical insights into SCI's acute, traumatic implications [24]. Their study in a canine model demonstrated that, immediately following injury, regions of the spinal cord that underwent acute trauma exhibit altered stiffness patterns. Notably, while increased stiffness was associated with the formation of changes in the injury environment, tissue during the early healing phase, intraoperative SWE also revealed that injured regions, in some cases, showed lower stiffness compared to uninjured areas. This finding may reflect tissue disruption and early edema rather than established fibrosis, though further investigation is needed to confirm this hypothesis. Al-Habib et al. conducted an in vivo study using SWE in dogs, providing foundational data on acute spinal cord elasticity changes under compression [25]. They reported that the mean spinal cord elasticity (SCE) in healthy dogs was 18.5 ± 7 kPa, but when a 13-mm balloon compression was applied, the stiffness increased significantly to 233 ± 73 kPa. These findings highlight the strong correlation between mechanical compression and increased spinal cord stiffness and reinforce the feasibility of SWE as a real-time diagnostic tool for accurately detecting spinal cord compression and predicting injury severity with enhanced fidelity.

Shajudeen et al. expanded on this by applying strain elastography (SE) in an acute SCI ex-vivo rabbit model, demonstrating that progressive increases in spinal stiffness correlated with worsening locomotor performance, as assessed by the Basso, Beattie, Bresnahan (BBB) locomotor scale [26]. Their statistical analysis showed significant differences in axial normal strain values between intact and fractured vertebrae, supporting the reliability of SE in detecting spinal fractures. While this may have implications for assessing underlying spinal cord effects, the study primarily focused on fracture identification rather than direct evaluation of cord damage, necessitating further investigation. Similarly, Tang et al. utilized strain elastography in a rabbit model of acute SCI and reported significant differences in strain ratios between paralyzed and non-paralyzed rabbits [27]. Their study showed that higher strain ratios were strongly correlated with spinal cord myelomalacia and histologic evidence of tissue disruption. Mechanical testing validation demonstrated that injured spinal cord regions had lower stiffness than non-injured regions, as indicated by differences in strain ratio and Young’s modulus. This relationship aligns with expected biomechanical properties, reinforcing the ability of elastography to quantify stiffness variations in spinal cord injury. These findings highlight that SWE may serve as an early biomarker for SCI severity by providing a real-time, non-invasive measure of tissue integrity. However, its current application requires a laminectomy for intraoperative use, and significant hurdles remain for non-invasive translation to human patients due to greater spinal cord depth.

These studies demonstrate that both SWE and SE provide real-time, intra-operative measurements of tissue stiffness that could prove critical in the immediate evaluation of traumatic SCI. By enabling rapid assessment of biomechanical changes, these modalities, once reliable, may improve early diagnosis, facilitate emergency treatment decisions, and help predict functional outcomes in acute spinal cord injuries.

### Chronic spinal cord compression and injury

#### Clinical studies

Chronic spinal cord compression from degenerative spinal conditions as well as extramedullary tumors triggers a cascade of microvascular, inflammatory, and fibrotic changes that can result in long-term neurological deficits if left untreated [28]. Unlike the acute setting, chronic compression involves a gradual evolution of tissue changes that can be monitored over time. SWE has emerged as a promising tool for assessing the biomechanical state of the spinal cord and potentially predicting clinical outcomes after surgical decompression. [25]

Al-Habib et al*.* conducted a study evaluating spinal cord elasticity (SCE) in patients with cervicothoracic myelopathy from chronic compressive lesions either from degenerative spine disease or extramedullary tumors [19]. Their results showed that intra-operative SWE values were significantly elevated in the compressed spinal cord, with a median elasticity of 93.84 kPa (IQR 75.27–121.75 kPa) in the spinal cords before resection of the compressive lesion versus 9.35 kPa (IQR 6.95–11.22 kPa) in decompressed patients (DCG). Following decompression surgery, SWE values significantly decreased in the compressed spinal cord group, and the degree of reduction in SWE values correlated with neurological improvement.

These findings underscore the potential of SWE not only as a diagnostic modality but also as a prognostic tool that can provide insight on the recoverability of the spinal cord after chronic compression from various pathologies. Based on the findings from this study, we propose that SWE could be integrated into several aspects of intraoperative imaging and possibly post-operative monitoring. Though current ultrasound imaging technology limits the ability to evaluate the spinal cord transcutaneously, the theoretical application of transcutaneous SWE to track spinal cord fibrosis development and assess decompression success could become a valuable imaging modality that could be performed in the clinic setting by an experienced sonographer. By establishing baseline stiffness thresholds and tracking their progress, clinicians can more accurately identify patients at higher risk for persistent deficits, enabling timely interventions that could ultimately optimize recovery.

### Comparisons to other modalities

While MRI and CT remain the gold standard for preoperative spinal evaluation, these modalities are primarily structural and do not assess real-time biomechanical properties of spinal tissues [29]. In contrast, USE provides a biomechanical assessment of tissue stiffness, offering additional information that may complement traditional imaging techniques, particularly in intraoperative and postoperative settings.

MRE is a non-invasive imaging method that quantifies tissue stiffness using low-frequency mechanical waves, producing high-resolution stiffness maps of deep tissues [17]. This highlights a potential need for additional modalities to offer a more comprehensive view of spinal cord elasticity than USE alone, which is limited by ultrasound signal attenuation. However, MRE has several drawbacks, including longer acquisition times, which make it less suitable for intraoperative use, higher costs and specialized equipment, which limits its accessibility, and intense motion artifacts, which can affect spinal cord stiffness measurements and require extensive post-processing. In contrast, USE provides practically real-time stiffness assessments, is portable, and is already integrated into many modern ultrasound systems, making it ideal for intraoperative applications. However, its depth penetration remains a limitation, as low frequency ultrasound probes suited for general deep imaging tend to have worse resolution than MRE. Other imaging modalities, such as CEUS and spinal angiography, offer critical insights into spinal cord perfusion and ischemic changes [3]. However, while CEUS enhances vascular imaging, it does not provide tissue elasticity data, making it complementary to USE but not a replacement. Similarly, standard CT and MRI provide excellent anatomical detail, allowing for the identification of spinal stenosis, disc herniations, and vertebral fractures, but they cannot assess mechanical integrity.

A potential hybrid approach using MRE and USE could optimize spinal pathology assessment. MRE could provide preoperative whole-spinal cord stiffness maps, allowing for deeper tissue analysis. At the same time, USE could serve as a real-time intraoperative adjunct, allowing surgeons to monitor dynamic changes in tissue stiffness during procedures. Future research should explore how integrating these modalities can improve diagnostic precision across preoperative, intraoperative, and postoperative settings, potentially enhancing treatment planning and surgical outcomes.

### Technical considerations and future outlook

Despite its promise, several technical and practical barriers must be addressed for the widespread clinical adoption of spinal USE. A primary challenge is acoustic access; the vertebral column creates significant acoustic shadowing, currently limiting USE to intraoperative applications where a laminectomy provides a direct acoustic window [30, 31]. The future development of specialized transducers capable of navigating inter-laminar spaces or novel processing algorithms to reduce osseous artifacts will be necessary for any potential non-invasive application.

Furthermore, while SWE is less operator-dependent than SE, measurement reliability is still influenced by factors such as transducer pressure, probe angle relative to the cord, and the presence of cerebrospinal fluid, all of which can affect shear wave propagation [12]. Standardized acquisition protocols, potentially guided by machine learning-based quality feedback systems, will be essential for ensuring reproducibility. The physical form factor of current probes can also be challenging for intraoperative use, as their geometry may not be compatible with the limited access of minimally invasive surgical corridors [7, 21]. Continued innovation in miniaturized, high-frequency transducers is therefore critical to enhance the current abilities of USE [30, 32]. Addressing these ultrasound-specific challenges through a collaborative effort between engineers and clinicians will be the key to translating this technology from a promising concept into a standard of care.

### Limitations

This systematic review has several important limitations that must be acknowledged when interpreting its findings. The primary limitation is the scarcity and heterogeneity of the available literature. Our comprehensive search yielded only seven studies that met the inclusion criteria, underscoring that USE for spinal pathology is still a nascent field. This small body of evidence consists of a mix of three clinical studies and four preclinical animal models, with varied methodologies, small sample sizes, and different pathological focuses. This significant heterogeneity precluded any form of quantitative statistical meta-analysis and limits the generalizability of the conclusions drawn.

Second, our search strategy had inherent limitations. While our preliminary investigation of the Scopus database and secondary sources revealed significant overlap, their formal exclusion from the final search strategy may have led to the omission of some relevant studies. Furthermore, the search was restricted to articles published in English, which may have introduced a language and selection bias.

Finally, there is a potential for publication bias, as is common in reviews of emerging technologies, where studies with positive or significant findings are more likely to be published than those with null or negative results. While the ROBINS-I tool was used to assess the quality of the included articles, the inherent risk of bias in the primary non-randomized studies cannot be fully eliminated and may influence the interpretation of their outcomes.

## Conclusion

Ultrasound elastography enhances the assessment of spinal cord pathology by providing real-time, quantitative measurements of tissue stiffness, addressing a key limitation of conventional structural imaging. This systematic review found that USE demonstrates significant diagnostic and prognostic utility across a range of conditions, including tethered cord syndrome, spinal tumors, and spinal cord injury. Moreover, the evidence confirms the value of intraoperative USE for enhancing surgical guidance, offering immediate feedback to quantify the success of an intervention, and improving real-time surgical decision-making.

As a cost-effective adjunct to MRI and CT, USE provides a unique biomechanical perspective that can improve both preoperative evaluation and postoperative monitoring. However, for USE to reach its full potential, future research must focus on standardizing protocols to overcome challenges like operator familiarity and technological limitations such as depth penetration. Establishing quantifiable stiffness standards for various pathologies is essential for its broader adoption. With continued innovation in transducer technology, beamforming, and processing algorithms, USE is poised to transform spinal imaging, paving the way for safer, more precise surgeries and improved patient outcomes.

## Supplementary Information

Supplementary Material 1.

Supplementary Material 2.

## Data Availability

No datasets were generated or analysed during the current study.
