# Supplementary material for: Intraoperative elastography and spinal surgery: a systematic review of current and future applications in clinical and preclinical models
Source: Ultrasound J. 2025 Nov 19;17:59. doi: 10.1186/s13089-025-00462-0 (PMC12630497; doi:10.1186/s13089-025-00462-0)
Supplement: Supplementary file 1 — Supplementary Material 1. [file 13089_2025_462_MOESM1_ESM.docx]

# Supplemental Table 1. Literature Search Strategy Across Databases

| Database | Search Strategy | Results Retrieved |
| --- | --- | --- |
| PubMed | ("Elastography Imaging Techniques"[Mesh] OR "Elastography"[tiab] OR "Sonoelastography"[tiab] OR "Shear Wave Elastography"[tiab] OR "Strain Elastography"[tiab]) AND ("Spine"[Mesh] OR "Spinal Cord"[Mesh] OR "Spinal Cord Injuries"[Mesh] OR "Spine"[tiab] OR "Spinal"[tiab] OR "Spinal Cord"[tiab] OR "Vertebra*"[tiab] OR "Myelopathy"[tiab]) AND ("Neurosurgical Procedures"[Mesh] OR "Intraoperative Period"[Mesh] OR "Spinal Cord Neoplasms"[Mesh] OR "Surgery"[tiab] OR "Surgical"[tiab] OR "Intraoperative"[tiab] OR "Operative"[tiab] OR "Tumor*"[tiab] OR "Neoplasm*"[tiab] OR "Injury"[tiab] OR "Compression"[tiab] OR "Pathology"[tiab]) | 150 |
| Cochrane Library | ([mh "Elastography Imaging Techniques"] OR Elastography:ti,ab,kw OR Sonoelastography:ti,ab,kw OR "Shear Wave Elastography":ti,ab,kw) AND ([mh Spine] OR [mh "Spinal Cord"] OR Spine:ti,ab,kw OR "Spinal Cord":ti,ab,kw OR Vertebra*:ti,ab,kw) AND ([mh "Neurosurgical Procedures"] OR Surgery:ti,ab,kw OR Surgical:ti,ab,kw OR Intraoperative:ti,ab,kw OR Tumor*:ti,ab,kw) | 50 |
| Web of Science | TS=("Elastography" OR "Sonoelastography" OR "Shear Wave Elastography" OR "Strain Elastography") AND TS=("Spine" OR "Spinal" OR "Spinal Cord" OR "Vertebra*" OR "Myelopathy") AND TS=("Surgery" OR "Surgical" OR "Intraoperative" OR "Operative" OR "Tumor*" OR "Neoplasm*" OR "Injury" OR "Compression" OR "Pathology") | 120 |
