# Supplementary material for: Intraoperative elastography and spinal surgery: a systematic review of current and future applications in clinical and preclinical models
Source: Ultrasound J. 2025 Nov 19;17:59. doi: 10.1186/s13089-025-00462-0 (PMC12630497; doi:10.1186/s13089-025-00462-0)
Supplement: Supplementary file 2 — Supplementary Material 2. [file 13089_2025_462_MOESM2_ESM.docx]

| **Study** | **Bias due to Confounding** | **Bias in Selection of Participants** | **Bias in Classification of Interventions** | **Bias due to Deviations from Interventions** | **Bias due to Missing Data** | **Bias in Measurement of Outcomes** | **Bias in Selection of Reported Result** | **Overall Risk of Bias** |
| --- | --- | --- | --- | --- | --- | --- | --- | --- |
| Al-Habib et al. 2021^15^ | Low | Low | Low | Low | Low | Low | Low | **Low** |
| Al-Habib et al. 2018^23^ | Serious | Moderate | Low | Moderate | Low | Low | Serious | **Serious** |
| Almotairi et al. 2023^18^ | Low | Low | Low | Low | Low | Low | Moderate | **Moderate** |
| Kerensky et al. 2024^16^ | Low | Low | Low | Low | Low | Low | Low | **Low** |
| Prager et al. 2020^22^ | Low | Low | Low | Low | Low | Low | Low | **Low** |
| Shajudeen et al. 2019^24^ | Low | Low | Low | Low | Low | Low | Low | **Low** |
| Tang et al. 2023^25^ | Moderate | Low | Low | Low | Low | Low | Low | **Moderate** |

**Supplementary Table 1. Detailed risk of bias assessment for each included primary study.** The table presents the judgments for the seven domains of the ROBINS-I (Risk Of Bias In Non-randomized Studies - of Interventions) tool, along with the overall risk of bias.
